# Investigation of the Potential of Commercial and Wild *Passiflora* Seed Species as Stilbenes Sources

**DOI:** 10.1021/acs.jafc.5c00440

**Published:** 2025-06-06

**Authors:** Ana Paula Lourenção Zomer, Carina Alexandra Rodrigues, Eliza Mariane Rotta, Nilton Tadeu Vilela Junqueira, Oscar Oliveira Santos, Jesuí-Vergílio Visentainer, Liane Maldaner

**Affiliations:** † Chemistry Department, State University of Maringá (UEM), 87020-900 Maringá, PR, Brazil; ‡ Brazilian Agricultural Research Corporation, Embrapa Cerrados, 73310-970 Brasília, DF, Brazil

**Keywords:** seeds, *Passiflora*, piceatannol, resveratrol, μ-QuEChERS, UHPLC-MS/MS

## Abstract

Passion fruit seeds, a byproduct of juice processing, are rich in bioactive stilbenes with health-promoting properties. This study investigated piceatannol and resveratrol in seeds from four commercial and sixteen wild *Passiflora* species using the μ-QuEChERS method combined with UHPLC-MS/MS analysis. The method showed good analytical performance, with linearity (*R* ^2^ ≥ 0.991), LOQ ≤ 20 μg kg^–1^, and RSD < 11%. Piceatannol and resveratrol were found in 70 and 60% of the analyzed species, respectively. Piceatannol was found in significantly higher amounts (0.6–55.2 mg kg^–1^) in 95% of these species, with values up to 56 times greater than resveratrol (0.3–7.5 mg kg^–1^). The highest piceatannol amounts were observed in the wild species *P. longifilamentosa* (55.2 mg kg^–1^) and *P. edulis x P. caerulea* (44.7 mg kg^–1^). These findings highlight *Passiflora* seeds as a valuable natural source of piceatannol, supporting their potential applications in functional foods, cosmetics, and pharmaceutical products.

## Graphical abstract

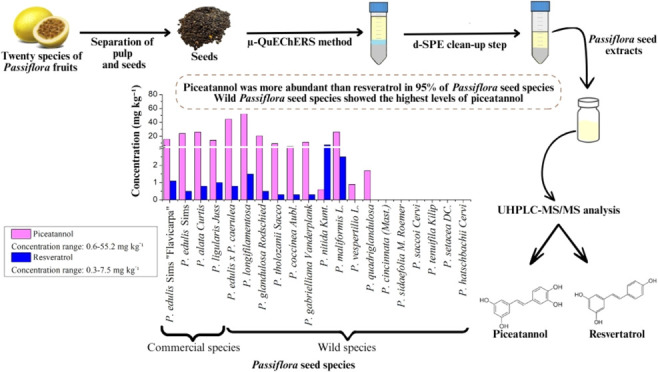

## 1 Introduction

The genus *Passiflora* comprises a wide range of species, with more than five hundred species distributed around the world, particularly in tropical and subtropical regions of the Americas.^1,2^ It is by far the most diverse and well-known genus of *Passifloraceae* family. Along with the diversity of species, *Passiflora* species have a wide range of applications, from ornamental and traditional medicine to pharmacological, cosmetic, and food uses, encompassing *Passiflora* flowers, leaves, roots, and fruits.^3,4^ For ornamental applications, *Passiflora* flowers from several species have been used due to their exotic beauty derived from unique shapes and colors.^5^ In traditional medicine, *Passiflora* leaves and roots, mainly from *P. edulis* and *P. incarnata* species, are widely used for their anxiolytic properties, helping to treat anxiety, insomnia, and depression.^3,5,6^ Regarding *Passiflora* fruits, known as passion fruits, the edible species granadilla (*P. ligularis Juss*), gulupa or purple passion fruit (*P. edulis “Sims”*), yellow or sour passion fruit (*P. edulis* Sims “Flavicarpa”), and sweet passion fruit (*P. alata Curtis*) are the most popular, with their pulp primarily used for both fresh consumption and juice production in the food industry.^2,6,7^

On the other hand, the peel and seeds which are byproducts in passion fruit juice production, have been highlighted in recent studies for containing high levels of bioactive compounds, with health-promoting properties, such as antioxidant,^8^ antidiabetic,^9^ anti-inflammatory,^10^ ultraviolet radiation (UV) skin damage prevention^11^ and anti-Alzheimer activity.^12^ Among the bioactive compounds already reported in passion fruits, phenolic compounds, such as anthocyanins and stilbenes, are the most frequently reported in peel and seeds, respectively.^6,13^

Although stilbenes are typically phenolic compounds associated with berry fruits, these compounds are also gaining recognition in passion fruit seeds due to the significant amounts of piceatannol and resveratrol found in extracts from some *Passiflora* seeds species, including *P. edulis* Sims “Flavicarpa”, *P. edulis* “Sims”, *P. alata Curtis* and *P. ligularis Juss*, with amounts reaching up to 2.2 and 0.1 mg g^–1^ respectively.^14^ Some lesser-known stilbenes, such as scirpusin B, piceid, and pinostilbene, have also been reported in passion fruit seeds, but in smaller amounts compared to piceatannol.^15,16^ In addition, the wide range of health-promoting properties attributed to stilbenes, such as anti-inflammatory, antitumor, UV blocker, and cellular antiphotoaging effects, may play an important role in expanding the application range of the whole passion fruit, as well as in promoting the sustainable use of byproducts from juice production.^17–19^

Despite the hundreds of *Passiflora* species distributed worldwide, only a small fraction, such as *P. edulis* ″Sims″, *P. edulis* Sims ″Flavicarpa″ and *P. incarnata*, have been the focus of most studies.^6^ As a result, many *Passiflora* species, including commercial and primarily wild species, have been scarcely studied or remain unexplored regarding their bioactive compounds and/or biological properties. Thus, there is a significant knowledge gap regarding potential applications of the whole passion fruit, particularly the seeds, which may act as promising sources of stilbenes for novel developments in pharmaceuticals and cosmetics. Furthermore, most published studies are based on conventional sample preparation techniques for bioactive compounds determination,^20,21^ while modern sample preparation techniques, which are more reliable and accurate, are still not widely used for phenolic compounds or stilbene determination in plant matrices.

Given the diversity of the *Passiflora* species and the lack of characterization of bioactive compounds in many of these species, there is a need for more comprehensive studies to investigate their bioactive potential and explore new applications. Therefore, the main aim of this work was to investigate stilbenes in *Passiflora* fruit seeds using a simple, rapid, reliable, and miniaturized sample preparation technique, the μ-QuEChERS method. To achieve this aim, twenty different *Passiflora* seed species were investigated for piceatannol and resveratrol, with their amounts accurately determined by ultrahigh performance liquid chromatography coupled to tandem mass spectrometry (UHPLC-MS/MS).

## 2 Materials and Methods

### 2.1 Chemicals, Reagents, and Standard Solutions

Piceatannol and resveratrol, with purities of above 98 and 99%, respectively, were purchased from Sigma-Aldrich (St. Louis, MO). HPLC-grade acetonitrile and methanol, sodium chloride, sodium acetate, anhydrous magnesium sulfate, and sodium citrate tribasic dihydrate were obtained from JT Baker (Edo. De Mexico, Mexico). HPLC-grade formic and acetic acids were purchased from Sigma-Aldrich (St. Louis, MO). Disodium hydrogen citrate sesquihydrate was acquired from Alpha Aesar (Ward Hill, MA). Primary secondary amine (PSA, 40 μm particle size) and graphitized carbon black (GCB) were obtained from Agilent Technologies, (Santa Clara, CA). Ultrapure water was obtained from a Milli-Q system (Millipore). Stock solutions of piceatannol and resveratrol standards (1000 mg L^–1^) were prepared in methanol, stored at −18 °C, and protected from light. Working solutions were prepared by appropriate dilutions of the stock solution in methanol, with concentrations ranging from 100 to 1 mg L^–1^.

### 2.2 Sampling

In this study, twenty different species of *Passiflora* seeds were investigated. *Passiflora ligulares Juss* and *Passiflora edulis* “Sims” were obtained from a local market in Maringá, Paraná, Brazil. *Passiflora alata Curtis*, *Passiflora setácea DC*, *Passiflora cincinnata (Mast.)*, *Passiflora edulis × P. caerulea*, *Passiflora longifilamentosa*, *Passiflora sidifolia M. Roemer*, *Passiflora tenuifila Kilip*, *Passiflora vespertilio L.*, *Passiflora saccoi Cervi*, *Passiflora nítida Kunt.*, *Passiflora gabrielliana vanderplank*, *Passiflora quadriglandulosa*, *Passiflora tholozanii Sacco*, *Passiflora coccínea Aubl.*, *Passiflora glandulosa Rodschied*, *Passiflora hatschbachii Cervi* and *Passiflora maliformis L.* were supplied by the Brazilian Agricultural Research Corporation (Empresa Brasileira de Pesquisa Agropecuária - Embrapa Cerrados), Brasília, Distrito Federal, Brazil. *Passiflora edulis* Sims “Flavicarpa” was obtained from Centrais de Abastecimento do Paraná S.A (CEASA/PR) in Maringá, Paraná, Brazil. The seeds were separated from the pulp using a fruit pulper (APITEC, DF-100), washed with running water, and dried in the shade at 27 ± 2 °C. The dried seeds were then ground in a Walita RI2106 blender, sieved through a 12-mesh sieve, vacuum-packed, and stored in a freezer at −18 °C until analysis.

### 2.3 Extraction Procedure: μ-QuEChERS Method

The μ-QuEChERS method developed in a previous study of Zomer et al.^22^ was adopted and further optimized to evaluate its applicability for determining piceatannol and resveratrol in passion fruit seeds. The optimized extraction conditions included examining the effect of the μ-QuEChERS method versions on the extraction amounts of the target stilbenes and the effect of the d-SPE cleanup sorbents on the removal of sample coextractives.

In brief, 0.625 g of passion fruit seeds were weighed and transferred to a 15 mL Falcon tube, followed by the addition of 1.875 mL of ultrapure water. After 30 min, 2.5 mL of acetonitrile (for the original and citrate versions) or acetonitrile acidified with 1%, acetic acid (for the acetate version) was added to the sample, and the mixture was vortexed (AP 56, Phoenix, Brazil) for 1 min. The partition step was then conducted by adding 1 g of magnesium sulfate (MgSO_4_) and 0.25 g of sodium chloride (NaCl) (original version), 1 g of MgSO_4_ and 0.25 g of sodium acetate (CH_3_COONa) (acetate version), or 1 g of MgSO_4_, 0.25 g of NaCl, 0.25 g of sodium citrate dihydrate (C_6_H_5_N_a3_O_7·_2H_2_O) and 0.125 g of sodium hydrogen citrate sesquihydrate (C_6_H_6_Na_2_O_7_·1.5H_2_O) (citrate version). The mixture was then vortexed for 1 min and centrifuged at 4000*g* for 10 min in a Harrier 18/80R centrifuge (Sanyo MSE, UK). After centrifugation, 1 mL of the supernatant was transferred to a new 15 mL Falcon tube for the d-SPE cleanup step. For this, 150 mg of MgSO_4_ and 25 mg of primary and secondary amine (PSA) were used as cleanup sorbents across all evaluated μ-QuEChERS method versions. Furthermore, the acetate version of the μ-QuEChERS method was also evaluated with the addition of 6.25 mg of graphitized carbon (GCB), resulting in a combination of 150 mg of MgSO_4_, 25 mg of PSA, and 6.25 mg of GCB for the cleanup step. All cleanup tubes were vortexed for 1 min and centrifuged at 4000*g* for 10 min. The supernatant was collected, filtered through polytetrafluoroethylene (PTFE) syringe filters (13 mm diameter, 0.22 μm pore size), and diluted 40-fold prior to chromatographic analysis. For sample extraction, μ-QuEChERS method was performed using the acetate version, with 150 mg of MgSO_4_, 25 mg of PSA, and 6.25 mg of CGB as d-SPE cleanup sorbents, following the same procedure described above. The experimental procedures followed for the optimization of the μ-QuEChERS method are summarized in a diagram, as shown in Figure 1.

**Figure 1:**
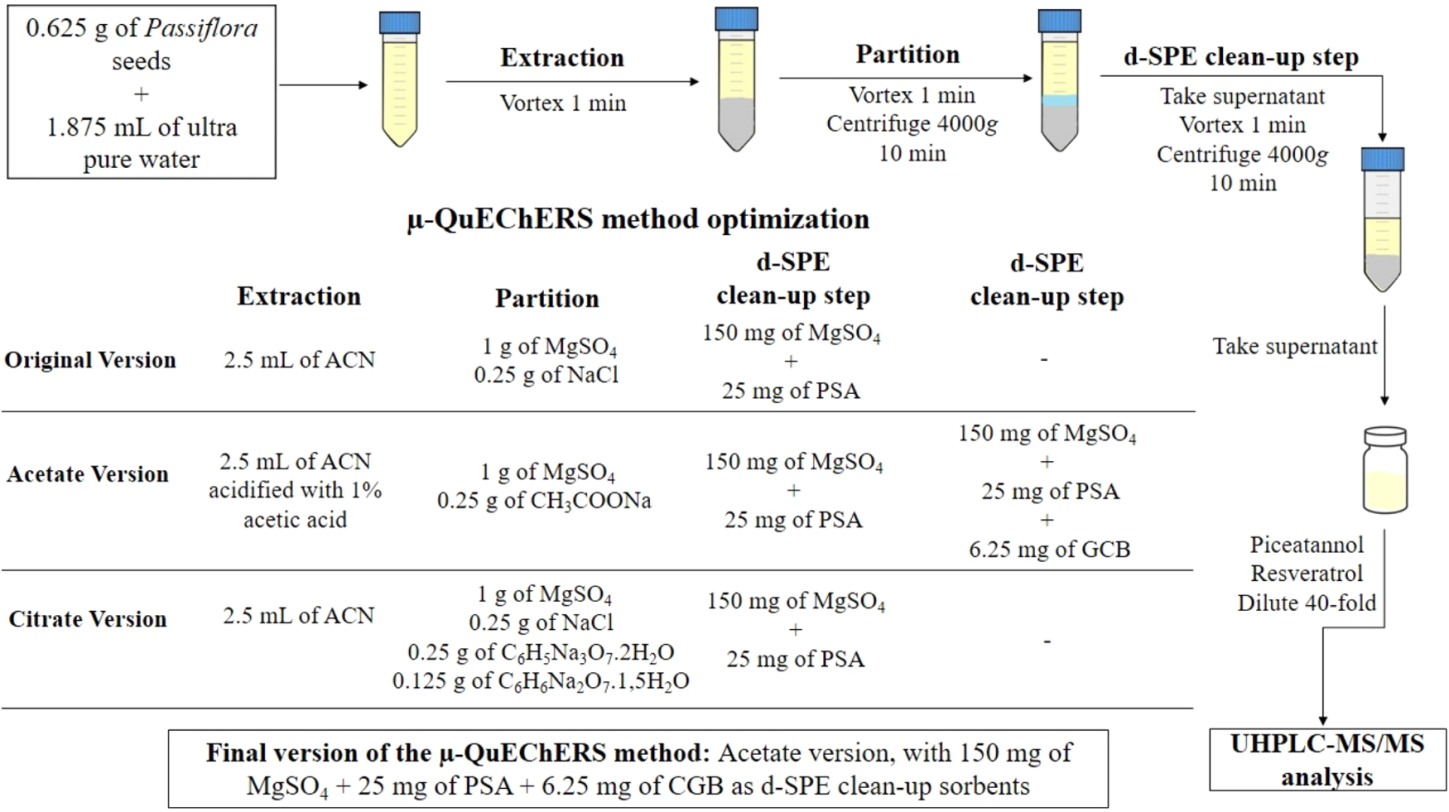
Flowchart illustrating the optimization steps of the μ-QuEChERS method for extracting piceatannol and resveratrol from *Passiflora* seeds.

### 2.4 Instrumentation and Chromatographic Conditions

Chromatographic analyses were performed by an Acquity H-CLASS UPLC (Waters, Milford, MA) coupled to a Xevo TQD triple-quadrupole mass spectrometer equipped with a Z spray source (Waters, Milford, MA). The chromatographic conditions and MS/MS mass spectrometer parameters described below were optimized to ensure optimal system performance for piceatannol and resveratrol analysis. Chromatographic separation was achieved using an Acquity UPLC BEH C18 1.7 μm column (50 × 2.1 mm i.d.) maintained at 25 °C. Chromatographic separation of piceatannol and resveratrol was performed using a binary mobile phase with (A) water (acidified with 0.1% formic acid) and (B) methanol in isocratic mode 50:50 v/v, a flow rate of 0.150 mL min^–1^, and a run time of 4 min. The sample injection volume was set to 1.50 μL.

The MS/MS mass spectrometer conditions were configured with the following parameters: the electrospray ionization (ESI) source was operated in negative mode, with a capillary voltage of 3.0 Kv, extractor voltage 3.0 V, source temperature of 130 °C, and desolvation temperature of 550 °C. Cone gas flow (nitrogen) was 50 L/h, and desolvation gas (nitrogen) was 700 L/h. Argon (99.9%) from White Martins (Rio de Janeiro, Brazil) was used as the collision gas at a constant pressure of 3.00 × 10 ^–3^ mbar. MassLynx and QuanLynx software version 4.1 (Waters) were used for instrument control, data acquisition, and processing. Mass spectrometric details, including retention time, precursor ions, cone energy, collision energy, and product ions, are shown in Supporting Information (Table S1).

### 2.5 Quantitative Analysis

Analytical parameters of the μ-QuEChERS-UHPLC-MS/MS method were evaluated, including linearity, the limit of detection (LOD) and quantification (LOQ), precision, and matrix effect (ME). Linearity was assessed using the standard addition method, where extracts from twenty species of passion fruit seeds (*Passiflora* spp.) were enriched with standard solutions at six concentration levels. Linearity was expressed in terms of the coefficient of determination (*R* ^2^), with an *R* ^2^ > 0.99 considered indicative of good linearity. The LOD and LOQ were defined as the analyte concentration that produced a chromatographic peak three and ten times, respectively, higher than the baseline noise in a not fortified sample chromatogram, after estimating the endogenous analyte amount. Method precision was calculated at the endogenous concentration of each target compound and expressed as the relative standard deviation (RSD). Matrix-effect values (% ME) were assessed by comparing the slopes of the analytical curves prepared in solvent with those prepared using the standard addition method on sample extracts (matrix) at identical concentration levels. ME values can be classified into three ranges: ≤ ± 20% (indicating no ME), ≥ ± 20% to ≤ ± 50% (indicating medium ME), and ≥ ± 50% (indicating strong ME).^23,24^ Additionally, ME values may be negative or positive, indicating signal suppression or enhancement, respectively.

### 2.6 Statistical Analysis

The experimental data presented in this study were obtained in triplicate and expressed as mean ± standard deviation (SD). Statistical analysis was performed using Assist software (version 7.7) to assess the differences among the experimental results. Data were analyzed using Tukey’s test, with statistical significance set at *p* < 0.05 for all comparisons.

## 3 Results and Discussion

### 3.1 Optimization of the μ-QuEChERS Method for Resveratrol and Piceatannol Extraction

The effect of the μ-QuEChERS method version on the extracted amounts of the target stilbenes, piceatannol and resveratrol, was assessed by comparing three versions of the QuEChERS method: original, acetate, and citrate. The original version is a nonbuffered method, meaning no pH adjustment is applied during extraction (pH depends on sample characteristics). In contrast, the acetate and citrate versions provide a buffering effect through the addition of sodium acetate (pH of 4.8) and a mixture of sodium citrate dihydrate and hydrogen citrate sesquihydrate (pH of 5.0–5.5), respectively.

For both piceatannol and resveratrol, the extracted amounts showed similar peak area percentages across all three μ-QuEChERS method versions, with no statistically significant differences (Figure 2). This result suggests that any version could be suitable. However, the μ-QuEChERS method will be applied to extract piceatannol and resveratrol from a wide range of *Passiflora* seed species with pH values ranging from 4.73 to 6.68 (Figure 3). Therefore, selecting a μ-QuEChERS method version that suits all the passion fruit seed species under study is crucial. Given that phenolic compounds, including the target stilbenes, are more stable under weakly acidic conditions, the acetate version, with its lower pH, was chosen.

**Figure 2:**
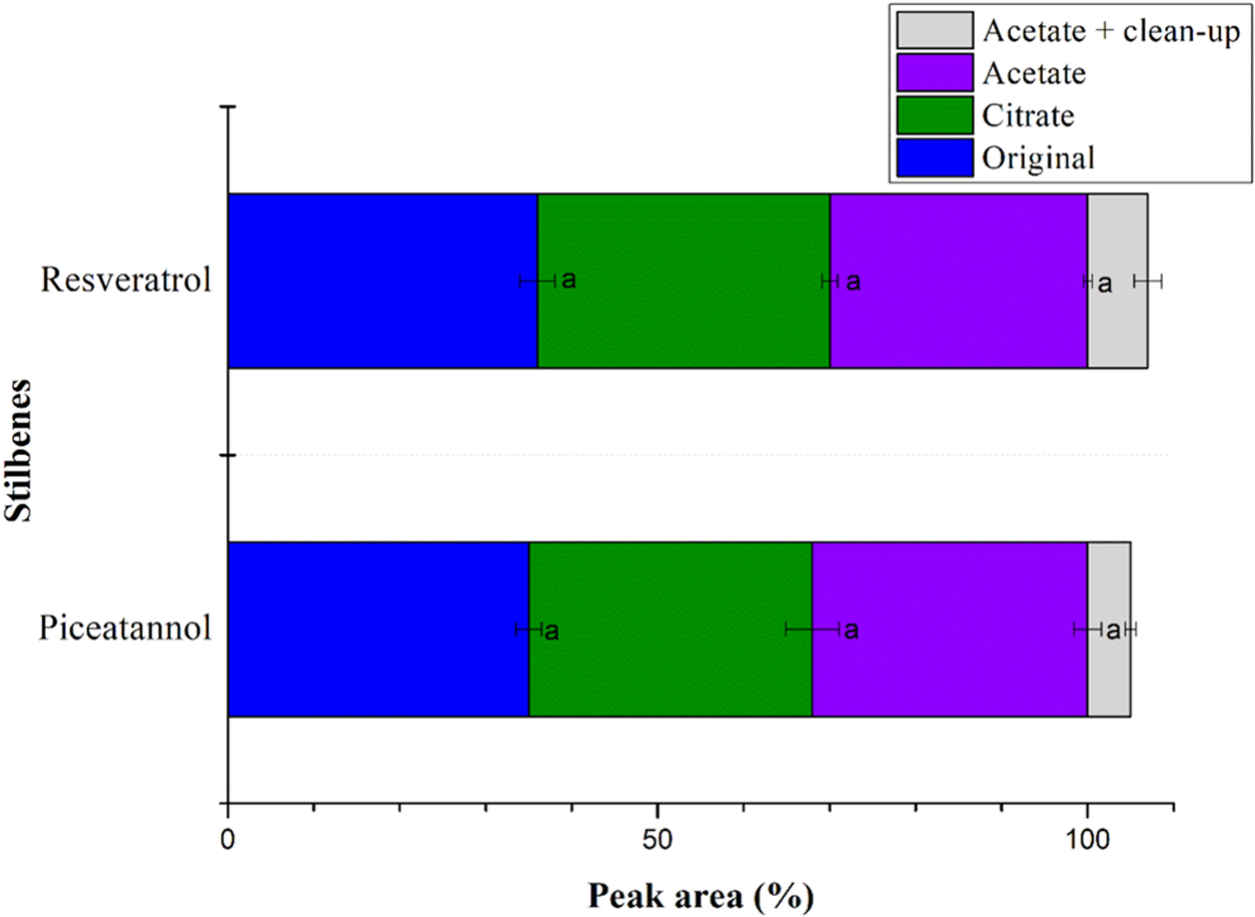
Evaluation of the original, acetate, and citrate μ-QuEChERS method versions for extracting piceatannol and resveratrol from *Passiflora* seeds. Data represents the mean peak area normalized to 100% across the μ-QuEChERS versions for each stilbene (*n* = 3). The μ-QuEChERS version followed by the same letter did not differ statistically from each other using the Tukey test (*p* < 0.05).

**Figure 3:**
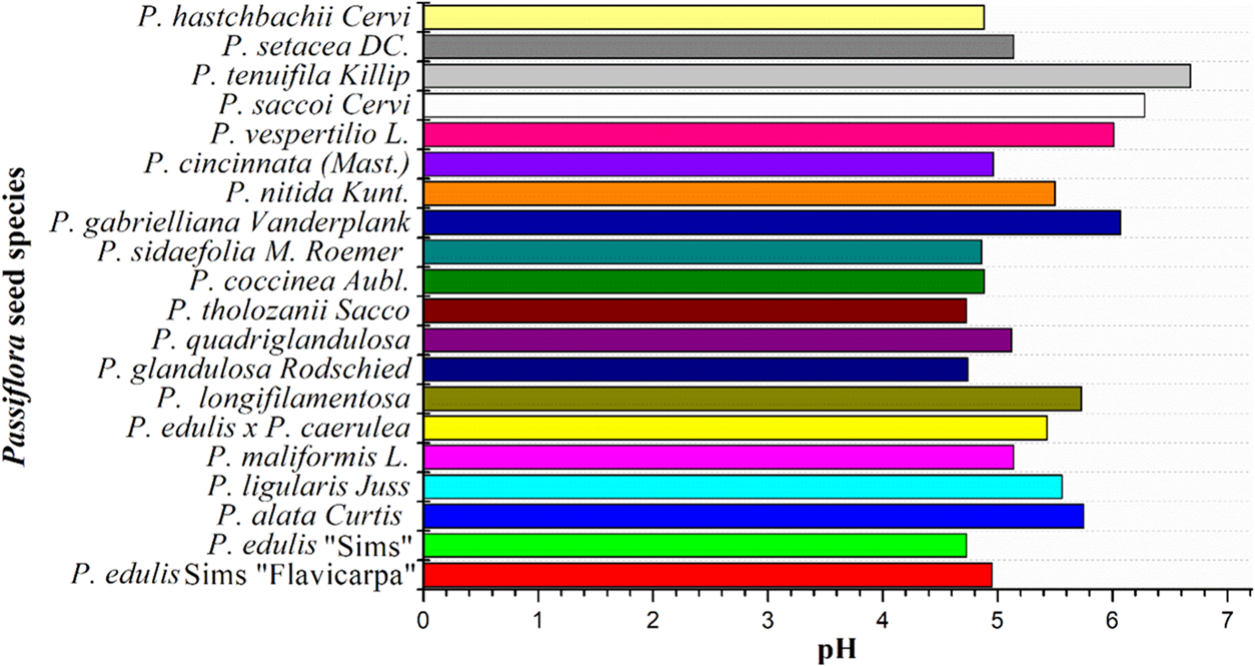
pH values of *Passiflora* seed extracts from the twenty species evaluated.

The influence of adding the combination of 25 mg of PSA and 6.25 mg of CGB in the d-SPE cleanup step was then investigated. First, the extracted amounts of piceatannol and resveratrol obtained with the acetate μ-QuEChERS method version and a d-SPE cleanup step using 25 mg of PSA and 6.25 mg of CGB were compared to the extracted amounts obtained with only 25 mg of PSA. As shown in Figure 2, when the d-SPE cleanup step was carried out using the combination of PSA and CGB, the extracted amounts increased by 5 and 7% for piceatannol and resveratrol, respectively, aligning with the findings of Zomer et al.^22^ Next, the effectiveness of the d-SPE cleanup step using 25 mg of PSA and 6.25 mg of CGB in removing interfering compounds was evaluated by calculating matrix effect values (% ME) for the seed extracts of each *Passiflora* species under study (Figure 4). Among the twenty passion fruit seed species evaluated, 70 and 85% showed matrix effect values of <± 20% for piceatannol and resveratrol, respectively, indicating no matrix effect. Extracts from *P. setácea DC.*, *P. cincinnata (Mast.)*, *P. tenuifila Kilip*, *P. saccoi Cervi*, and *P. hatschbachii Cervi* showed slightly higher matrix effect values, classified as medium matrix effect (>± 20% and <± 50%). The increase in matrix effect could be attributed to the reddish color of the seed extracts from these species. Based on these results, the d-SPE cleanup step using 25 mg of PSA and 6.25 mg of CGB was selected for sample analysis.

**Figure 4:**
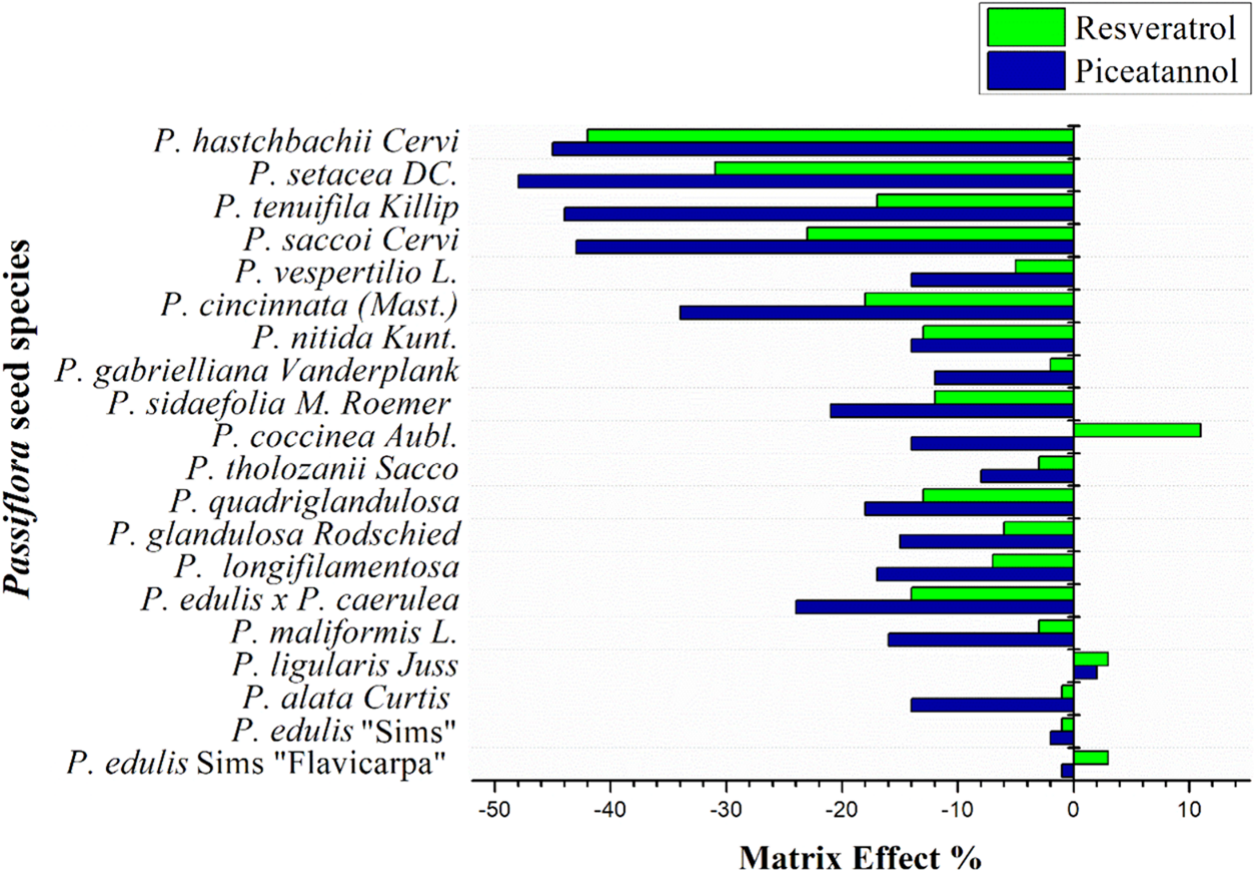
Evaluation of the d-SPE cleanup step efficiency in terms of matrix effect (% ME) for *Passiflora* seed extracts from the twenty species studied.

### 3.2 Application of the μ-QuEChERS-UHPLC-MS/MS Method for Piceatannol and Resveratrol Determination in *Passiflora* Seeds

After optimizing the μ-QuEChERS method, the best conditions were selected to evaluate the method analytical performance. The analytical parameters of the optimized method are summarized in Table 1. The chromatograms and ESI(−)-MS/MS profiles of piceatannol and resveratrol standards, as well as chromatograms of passion fruit seed extracts from the species with the highest amounts of piceatannol and resveratrol are shown in the Supporting Information (Figure S1). Analytical curves were generated using six standards by the standard addition method, covering concentration ranges of 13 to 1500 μg kg^–1^ for piceatannol and 3 to 500 μg kg^–1^ for resveratrol. Both compounds demonstrated good linearity across these concentration ranges, with coefficients of determination (R^2^) exceeding 0.991. The compounds exhibited LOD and LOQ of 4 to 6 μg kg^–1^ and 13 to 20 μg kg^–1^ for piceatannol, and 1 to 2 μg kg^–1^ and 3 to 6 μg kg^–1^ for resveratrol. These results indicate that the μ-QuEChERS-UHPLC-MS/MS method has suitable sensitivity for the concentration levels of piceatannol and resveratrol commonly found in plant matrices. Additionally, the μ-QuEChERS-UHPLC-MS/MS method demonstrated RSD values below 11% for the quantification of resveratrol and piceatannol, and matrix-effect values < ± 20% for at least 70% of the evaluated passion fruit seed species (see discussion in Section 3.1). Accordingly, the μ-QuEChERS-UHPLC-MS/MS method demonstrated suitable analytical performance for accurately determining piceatannol and resveratrol in *Passiflora* seeds. Comparatively, the analytical performance of the μ-QuEChERS-UHPLC-MS/MS method was similar to that reported in previous studies by our research group, which also focused on the determination of phenolic compounds in plant matrices using the QuEChERS method,^25–27^ and was superior to conventional sample preparation techniques for the quantitative determination of resveratrol and piceatannol in plant matrices.^28–30^

**Table 1:** Analytical Performance of the μ-QuEChERS-UHPLC-MS/MS Method for Determining Piceatannol and Resveratrol in Seeds of twenty *Passiflora* Species

			linear regression			
*Passiflora* seed species	compounds	linear range (μg kg^–1^)	*y* = *ax* + *b*	(*R* ^2^)^a^	LOD^b^/(μg kg^‑1^)	LOQ^c^/(μg kg^–1^)
*P. edulis* Sims “Flavicarpa”	Piceatannol	13–1500	*y* = 41.028*x* + 19132	0.9941	4	13
	Resveratrol	6–500	*y* = 41.005*x* + 971.09	0.9958	2	6
*P. edulis* “Sims”	Piceatannol	13–1500	*y* = 40.334*x* + 24869	0.9941	4	13
	Resveratrol	6–500	*y* = 39.608*x* + 459.22	0.9992	2	6
*P. alata Curtis*	Piceatannol	13–1500	*y* = 35.473*x* + 25231	0.9960	4	13
	Resveratrol	6–500	*y* = 39.172*x* + 704.57	0.9999	2	6
*P. ligularis Juss*	Piceatannol	13–1500	*y* = 42.223*x* + 17385	0.9914	4	13
	Resveratrol	6–500	*y* = 40.994*x* + 1030.9	0.9973	2	6
*P. maliformis L.*	Piceatannol	13–1500	*y* = 34.491*x* + 24253	0.9977	4	13
	Resveratrol	6–500	*y* = 38.385*x* + 2652.4	0.9998	2	6
*P. edulis x P. caerulea*	Piceatannol	13–1500	*y* = 27.975*x* + 34826	0.9937	4	13
	Resveratrol	6–500	*y* = 30.467*x* + 844.85	0.9961	2	6
*P. longifilamentosa*	Piceatannol	13–1500	*y* = 34.389*x* + 51991	0.9928	4	13
	Resveratrol	6–500	*y* = 36.985*x* + 1511.1	0.9998	2	6
*P. glandulosa Rodschied*	Piceatannol	20–1500	*y* = 31.255*x* + 18458	0.9911	6	20
	Resveratrol	6–500	*y* = 33.041*x* + 507.21	0.9993	2	6
*P. quadriglandulosa*	Piceatannol	13–1500	*y* = 30.141*x* + 1764.9	0.9993	4	13
	Resveratrol	6–500	*y* = 30.659*x* + 281.35	0.9966	2	6
*P. tholozanii Sacco*	Piceatannol	20–1500	*y* = 33.945*x* + 7203.9	0.9951	6	20
	Resveratrol	3–500	*y* = 34.266*x* + 229.25	0.9975	1	3
*P. coccinea Aubl.*	Piceatannol	20–1500	*y* = 31.568*x* + 6858.4	0.9994	6	20
	Resveratrol	3–500	*y* = 32.502*x* + 110.22	0.9930	1	3
*P. sidaefolia M. Roemer*	Piceatannol	13–1500	*y* = 29.092*x* + 161.49	0.9999	4	13
	Resveratrol	6–500	*y* = 31.021*x* + 180.23	0.9989	2	6
*P. nitida Kunt.*	Piceatannol	13–1500	*y* = 31.515*x* + 741.62	0.9988	4	13
	Resveratrol	6–500	*y* = 30.718*x* + 6193.5	0.9983	2	6
*P. gabrielliana Vanderplank*	Piceatannol	13–1500	*y* = 36.118*x* + 13128	0.9978	4	13
	Resveratrol	3–500	*y* = 39.119*x* + 387.11	0.9996	1	3
*P. cincinnata (Mast.)*	Piceatannol	13–1500	*y* = 24.081*x* + 326.26	0.9969	4	13
	Resveratrol	6–500	*y* = 28.736*x* + 215.09	0.9988	2	6
*P. vespertilio L.*	Piceatannol	20–1500	*y* = 31.584*x* + 667.56	0.9987	6	20
	Resveratrol	6–500	*y* = 33.65*x* + 29.245	0.9997	2	6
*P. saccoi Cervi*	Piceatannol	13–1500	*y* = 20.844*x* – 129.24	0.9973	4	13
	Resveratrol	6–500	*y* = 27.158*x* + 303.09	0.9952	2	6
*P. tenuifila Kilip*	Piceatannol	13–1500	*y* = 20.642*x* + 240.61	0.9984	4	13
	Resveratrol	6–500	*y* = 29.162*x* – 22.539	0.9933	2	6
*P. setacea DC.*	Piceatannol	13–1500	*y* = 19.242*x* + 421.65	0.9972	4	13
	Resveratrol	6–500	*y* = 24.419*x* + 349.81	0.9964	2	6
*P. hatschbachii Cervi*	Piceatannol	13–1500	*y* = 20.132*x* + 781.24	0.9953	4	13
	Resveratrol	6–500	*y* = 20.536*x* + 167.88	0.9989	2	6

a *R* ^2^: determination coefficient.

b LOD: limit of detection.

c LOQ: limit of quantification.

The established μ-QuEChERS-UHPLC-MS/MS method was applied for the determination of piceatannol and resveratrol in 20 *Passiflora* seed species. The selected *Passiflora* species include four well-known species worldwide and 16 wild species, all of which are edible. The amounts of resveratrol and piceatannol found in these passion fruit seed extracts are summarized in Table 2.

**Table 2:** Amounts of Piceatannol and Resveratrol Found in the Seeds of the twenty Evaluated *Passiflora* Species

*Passiflora* seed species		piceatannol (mg kg^–1^)	resveratrol (mg kg^‑1^)
commercial species	*P. edulis* Sims “Flavicarpa”	15.8 ^f^ ± 2.0	1.1 ^d^ ± 0.1
	*P. edulis* “Sims”	23.9 ^d^ ± 1.0	0.5 ^fg^ ± 0.2
	*P. alata Curtis*	26.1 ^c^ ± 1.5	0.8 ^ef^ ± 0.1
	*P. ligularis Juss*	14.3 ^f^ ± 1.8	1.0 ^d^ ± 0.1
			
wild species	*P. edulis × P. caerulea*	44.7 ^b^ ± 0.6	0.8 ^de^ ± 0.1
	*P. longifilamentosa*	55.2 ^a^ ± 0.4	1.5 ^c^ ± 0.3
	*P. glandulosa Rodschied*	20.6 ^e^ ± 0.8	0.5 ^fg^ ± 0.1
	*P. tholozanii Sacco*	9.4 ^h^ ± 1.4	0.3 ^g^ ± 0.1
	*P. coccínea Aubl.*	5.9 ^i^ ± 1.3	0.3 ^g^ ± 0.1
	*P. gabrielliana Vanderplank*	11.6 ^g^ ± 2.6	0.3 ^g^ ± 0.1
	*P. nitida Kunt.*	0.6 ^j^ ± 1.2	7.5^a^ ± 0.4
	*P. maliformis L.*	26.4 ^c^ ± 0.9	2.5^b^ ± 0.1
	*P. vespertilio L.*	0.9 ^j^ ± 2.9	<LOD
	*P. quadriglandulosa*	1.7 ^j^ ± 2.4	<LOD
	*P. cincinnata (Mast.)*	<LOD	<LOD
	*P. sidaefolia M. Roemer*	<LOD	<LOD
	*P. saccoi Cervi*	<LOD	<LOD
	*P. tenuifila Kilip*	<LOD	<LOD
	*P. setacea DC.*	<LOD	<LOD
	*P. hatschbachii Cervi*	<LOD	<LOD

Piceatannol and resveratrol were found in 70% and 60% of the analyzed passion fruit seed species, respectively, with amounts ranging from 0.6 to 55.2 mg kg^–1^ for piceatannol and 0.3 to 7.5 mg kg^–1^ for resveratrol. In 95% of the *Passiflora* seed species evaluated, piceatannol amounts were higher than those of resveratrol, suggesting that *Passiflora* seeds are a richer source of piceatannol. In particular, seeds from *P. edulis x P. caerulea*, *P. edulis “Sims”*, *P. longifilamentosa*, and *P. alata Curtis* showed piceatannol amounts approximately 56, 48, 37, and 33 times higher than resveratrol, respectively. Although all well-known *Passiflora* species showed high piceatannol amounts, the highest amounts were found in the wild species, *P. longifilamentosa* (55.2 mg kg^–1^) and *P. edulis x P. caerulea* (44.7 mg kg^–1^).

Table 3 presents a summary of published studies on the quantitative determination of piceatannol and resveratrol in passion fruit seeds. Except for our previous study,^22^ which included four widely known *Passiflora* species, other reports mainly focus on *Passiflora edulis*, the most economically important and widely distributed species. Furthermore, the majority of these studies evaluated only amounts of piceatannol. These studies are therefore highly relevant, as they demonstrate that the seeds of cultivated commercially important *Passiflora* species are rich in piceatannol. Among the studies that also assessed resveratrol, the data show that resveratrol is present in the seeds, although typically in lower amounts. However, to the best of our knowledge, no previous studies have investigated the amounts of piceatannol and resveratrol in wild *Passiflora* species. Given the large number of *Passiflora* species worldwide and the lack of bioactive compounds characterization in many of them, there is a knowledge gap regarding the potential health-promoting properties of several *Passiflora* species. Thus, the findings of this study contribute significantly to a more comprehensive characterization of passion fruit seeds, from well-known species to not yet studied wild species, in terms of identifying and quantifying the main stilbenes commonly reported for passion fruit seeds. This study evaluated four well-known commercial cultivated *Passiflora* species and 16 wild species. For all four commercially cultivated species, both piceatannol and resveratrol were found in the seed extracts. On the other hand, although resveratrol and piceatannol were absent in some wild seed extracts, other wild species proved to be much richer sources of these compounds compared to the well-known commercially cultivated species.

**Table 3:** Comparison of Studies on the Quantitative Determination of Piceatannol and Resveratrol in Passion Fruit Seeds (*Passiflora* spp.)

reference	sample	*Passiflora* seed species	concentration of piceatannol and resveratrol
present study	*in natura* passion fruit seeds	*Passiflora edulis* Sims “Flavicarpa”	
		*Passiflora ligulares Juss*	
		*Passiflora edulis* “Sims”	
		*Passiflora alata Curtis*	
		*Passiflora edulis x P. caerulea*	
		*Passiflora longifilamentosa*	
		*Passiflora vespertilio L.*	
		*Passiflora nitida Kunt.*	PIC: 0.6 to 55.2 mg kg^–1^
		*Passiflora gabrielliana Vanderplank*	RES: 0.3 to 7.5 mg kg^–1^
		*Passiflora quadriglandulosa*	
		*Passiflora tholozanii Sacco*	
		*Passiflora coccínea Aubl.*	
		*Passiflora glandulosa Rodschied*	
		*Passiflora maliformis L.*	
			
^22^	*in natura* passion fruit seeds	*Passiflora edulis* Sims “Flavicarpa”	PIC: 7.5 to 20.8 mg kg^–1^
		*Passiflora ligulares Juss*	
		*Passiflora edulis* “Sims”	
		*Passiflora alata Curtis*	
			
^14^	defatted and lyophilized passion fruit seeds	*Passiflora edulis*	PIC: 2.2 mg g^–1^
			RES: 0.1 mg g^–1^
			
^15^	lyophilized passion fruit seeds	*Passiflora edulis*	PIC: 104.5 μg mg^–1^
			RES: 0.082 mg g^–1^
			
^28^	*in natura* passion fruit seeds	*Passiflora edulis*	PIC: 9–12 μg mL^–1^
			RES: 10–33 μg mL^–1^
			
^38^	defatted passion fruit seeds	*Passiflora edulis* “Sims”	PIC: 28.9 mg 300 mg^–1^ of seeds extract
			
^40^	lyophilized passion fruit seeds	*Passiflora edulis* “Sims”	PIC: 3.68 100 g^–1^ seeds
			
^39^	lyophilized passion fruit seeds	*Passiflora edulis*	PIC: 660 μM in 160 mg mL^–1^ of extract
			RES: 12 μM in 160 mg mL^–1^ of extract
			
^16^	lyophilized passion fruit seeds	*Passiflora edulis*	PIC: 570 mg in 100 g of dried seeds
			
^29^	defatted and nondefatted passion fruit bagasse	*P. edulis sp.*	PIC: 1.529–18.590 mg g^–1^ of bagasse
			
^41^	*in natura* passion fruit byproducts (seed and pulp)	*Passiflora edulis*	PIC: 0.128–1.81 mg g^–1^ of dried passion fruit byproducts extract
			
^42^	lyophilized passion fruit seeds	*Passiflora edulis*	PIC: 85.4 μg mg^–1^
			
^43^	defatted passion fruit seeds	*Passiflora edulis* Sims var. flavicarpa	PIC: 1403.17 mg 100 g^–1^ of defatted seeds
			
^34^	lyophilized passion fruit seeds	*Passiflora edulis*	PIC: 94.9 μg mg^–1^
			
^44^	defatted passion fruit seeds	*Passiflora edulis “*Sims”	PIC: 16 mg g^–1^
			
^45^	lyophilized passion fruit seeds	*Passiflora edulis*	PIC: 4.742 mg g^–1^ of extract
			
^46^	defatted passion fruit seeds	*Passiflora edulis “*Sims”	PIC: 13.03–27.17 μg mg^–1^
			
^10^	lyophilized passion fruit bagasse	*Passiflora edulis “*Sims”	PIC: 31.65 mg g^–1^ of dry extract
			
^47^	*in natura* passion fruit (seeds, peel and pulp) immature, mature and ripe	*Passiflora edulis*	Immature, mature and ripe seeds
			RES: nd
			Immature, mature and ripe peel
			RES: 0.3–0.34 mg 100 g^–1^
			Immature, mature, and ripe pulp
			RES: nd–0.43 mg 100 g^–1^

PIC: piceatannol RES: resveratrol.

Stilbenes such as resveratrol and piceatannol are associated with various health-promoting properties, including antioxidant, anti-inflammatory, and anticancer activities,^17,19,31^ These biological activities have motivated numerous studies evaluating the beneficial physiological effects of *Passiflora* seed extracts rich in these compounds. Studies have shown that *Passiflora* seed extracts exhibit antiproliferative effects in human cancer cells;^15,32^ collagenase, elastase, and hyaluronidase inhibitory effects;^33^ antidiabetic effect;^34^ antioxidant activity;^35,36^ emollient activity;^37^ α-glucosidase inhibitory activity;^38^ antiaging activity;^11^ antiallergy activity;^39^ and antimicrobial activity.^36^

Based on the established biological activities for stilbenes and *Passiflora* seed extracts, along with the findings of our study, we expect that this research will help add value to passion fruit residues, particularly passion fruit seeds. Given the high amounts of piceatannol and resveratrol found in some of well-known and wild passion fruit seeds, this study will encourage the sustainable use of passion fruit seeds, including those from wild *Passiflora* species, and support future developments of these seeds as natural ingredients in functional foods, cosmetic and pharmaceutical products.

## Supplementary Material



## Notes

Mass spectrometric details, including retention time, precursor ions, cone energy, collision energy, and product ions, are shown in Table S1. The chromatograms and ESI(−)-MS/MS profiles of piceatannol and resveratrol standards, as well as chromatograms of passion fruit seed extracts from the species with the highest amounts of piceatannol and resveratrol, are shown in Figure S1 (PDF)

## Abbreviations and nomenclature

UHPLC-MS/MS: ultrahigh performance liquid chromatography coupled to tandem mass spectrometry
QuEChERS: acronym for Quick, Easy, Cheap, Effective, Rugged, and Safe
UV: ultraviolet radiation
PSA: primary secondary amine
GCB: graphitized carbon black
d-SPE: dispersive solid phase extraction
NaCl: sodium chloride
MgSO_4_: magnesium sulfate
CH_3_COONa: sodium acetate
C_6_H_5_N_a3_O_7_·2H_2_O: sodium citrate dehydrate
C_6_H_6_Na_2_O_7_·1,5H_2_O: sodium hydrogen citrate sesquihydrate
PTFE: polytetrafluoroethylene
UPLC: ultra performance liquid chromatography
MS/MS: tandem mass spectrometry
ESI: electrospray ionization
mg: milligram
min: minute
mL: milliliter
g: gram
kg: kilogram
L: liter
μ: micro
Kv: kilovolt
V: volt
LOD: limit of detection
LOQ: limit of quantification
ME: matrix effect
*R* ^2^: coefficient of determination
RSD: relative standard deviation
SD: standard deviation
