# Supplementary material for: Investigation of the Potential of Commercial and Wild Passiflora Seed Species as Stilbenes Sources
Source: J Agric Food Chem. 2025 Jun 6;73(24):15046–55. doi: 10.1021/acs.jafc.5c00440 (PMC12186530; doi:10.1021/acs.jafc.5c00440)
Supplement: Supplementary file 1 [file jf5c00440_si_001.pdf]

Investigation of the potential of commercial and wild *Passiflora* seed species as stilbenes sources

Ana Paula Lourenção Zomer[a], Carina Alexandra Rodrigues[a], Eliza Mariane Rotta[a], Nilton Tadeu Vilela Junqueira[b], Oscar Oliveira Santos[a], Jesuí-Vergílio Visentainer[a], Liane Maldaner[a,*]

[a] Chemistry Department, State University of Maringá (UEM), 87020-900, Maringá-PR, Brazil.

[b] Brazilian Agricultural Research Corporation, Embrapa Cerrados, 73310-970, Brasília-DF, Brazil

*** Corresponding author:**

Liane Maldaner

Department of Chemistry, State University of Maringá. Av. Colombo, 5790, Maringá - PR, CEP 87020-900, Brazil

Tel: + 55 (044) 3011-3659

E-mail: lianemaldaner@gmail.com

Table S1 - Selected ion transitions and instrumental parameters for the analysis of the compounds under study.

**Compounds**	**Retention time**	**Precursor ion (m/z)**	**Cone energy (V)**	**Collision energy (V)**	**SRM transition (m/z)[a]**
**Piceatannol**	1.25	242.0 $[M-H]^-$	47	28	242.0>201.1
				22	242.0>159.0
**Resveratrol**	1.64	226.0 $[M-H]^-$	45	19	226.0>185.0
				27	226.0>143.0

[a] The first transition of each compound was used for quantification and the second one for confirmation purposes.

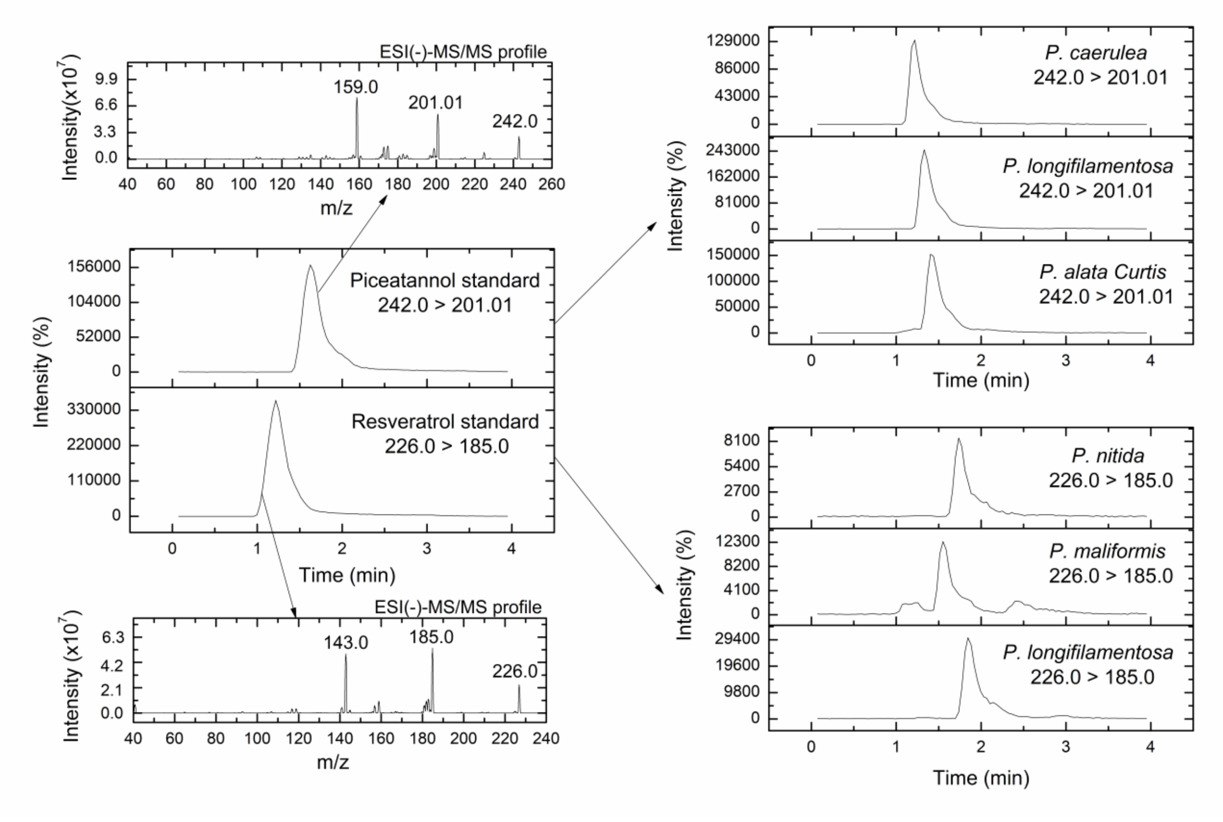

Figure S1 – Chromatograms and ESI(-)-MS/MS profiles of piceatannol and resveratrol standards, as well as chromatograms of passion fruit seed extracts from the species with the highest amounts of piceatannol and resveratrol. Chromatographic conditions are detailed in section 2.4.
